# Cortical glutamate, Glx, and total *N*-acetylaspartate: potential biomarkers of repetitive transcranial magnetic stimulation treatment response and outcomes in major depression

**DOI:** 10.1038/s41398-023-02715-9

**Published:** 2024-01-06

**Authors:** Meghan A. Gonsalves, Tara L. White, Jennifer Barredo, Marilena M. DeMayo, Emily DeLuca, Ashley D. Harris, Linda L. Carpenter

**Affiliations:** 1 Neuroscience Graduate Program, Brown University, Providence, RI USA; 2 Butler Hospital Neuromodulation Research Facility, Providence, RI USA; 3 Center of Biomedical Research Excellence (COBRE) for Neuromodulation, Butler Hospital, Providence, RI USA; 4 Center for Alcohol and Addiction Studies, Brown University, Providence, RI USA; 5 Department of Behavioral and Social Sciences, School of Public Health, Brown University, Providence, RI USA; 6 Carney Institute for Brain Sciences, Brown University, Providence, RI USA; 7 Department of Psychiatry and Human Behavior, Alpert Medical School, Brown University, Providence, RI USA; 8 Providence VA Medical Center, Providence, RI USA; 9 Clinical Neuroimaging Research Core, Brown University, Providence, RI USA; 10 Department of Radiology, University of Calgary, Calgary, AB Canada; 11 Alberta Children’s Hospital Research Institute, University of Calgary, Calgary, AB Canada; 12 Hotchkiss Brain Institute, University of Calgary, Calgary, AB Canada

**Keywords:** Predictive markers, Human behaviour, Depression, Physiology

## Abstract

Repetitive transcranial magnetic stimulation (rTMS) is an effective treatment for individuals with major depressive disorder (MDD) who have not improved with standard therapies. However, only 30–45% of patients respond to rTMS. Predicting response to rTMS will benefit both patients and providers in terms of prescribing and targeting treatment for maximum efficacy and directing resources, as individuals with lower likelihood of response could be redirected to more suitable treatment alternatives. In this exploratory study, our goal was to use proton magnetic resonance spectroscopy to examine how glutamate (Glu), Glx, and total *N*-acetylaspartate (tNAA) predict post-rTMS changes in overall MDD severity and symptoms, and treatment response. Metabolites were measured in a right dorsal anterior cingulate cortex voxel prior to a standard course of 10 Hz rTMS to the left DLPFC in 25 individuals with MDD. MDD severity and symptoms were evaluated via the Inventory of Depression Symptomatology Self-Report (IDS-SR). rTMS response was defined as ≥50% change in full-scale IDS-SR scores post treatment. Percent change in IDS-SR symptom domains were evaluated using principal component analysis and established subscales. Generalized linear and logistic regression models were used to evaluate the relationship between baseline Glu, Glx, and tNAA and outcomes while controlling for age and sex. Participants with baseline Glu and Glx levels in the lower range had greater percent change in full scale IDS-SR scores post-treatment (*p* < 0.001), as did tNAA (*p* = 0.007). Low glutamatergic metabolite levels also predicted greater percent change in mood/cognition symptoms (*p* ≤ 0.001). Low-range Glu, Glx, and tNAA were associated with greater improvement on the immuno-metabolic subscale (*p* ≤ 0.003). Baseline Glu predicted rTMS responder status (*p* = 0.025) and had an area under the receiving operating characteristic curve of 0.81 (*p* = 0.009), demonstrating excellent discriminative ability. Baseline Glu, Glx, and tNAA significantly predicted MDD improvement after rTMS; preliminary evidence also demonstrates metabolite association with symptom subdomain improvement post-rTMS. This work provides feasibility for a personalized medicine approach to rTMS treatment selection, with individuals with Glu levels in the lower range potentially being the best candidates.

## Introduction

Major depressive disorder (MDD) is a devastating neuropsychiatric illness that affects approximately 4% of individuals around the globe annually [1]. It is associated with increased mortality [2], poor health outcomes [3, 4], economic burden [5], heightened disability [6, 7], and decreased life satisfaction [8]. Failure to benefit from standard antidepressant treatments, such as psychotherapy and psychiatric medication, is common and approximately 33% of treated MDD patients exhibit persistent symptoms after adequate pharmacotherapy [9], a syndrome referred to as treatment-resistant depression (TRD) [10, 11].

One promising alternative treatment for TRD is repetitive transcranial magnetic stimulation (rTMS), a type of noninvasive brain stimulation [12, 13]. rTMS has been widely adopted in clinical practice and has proven effective for MDD [14]. Large registry data indicates that while most patients benefit from rTMS, about one-third of those with MDD don’t respond (i.e., achieve ≥ 50% decrease in depression scores) [15, 16]. Furthermore, rTMS therapy is costly and time-intensive, with treatments 5 days a week for 4–6 weeks [12]. Thus, identifying individuals most likely to respond to rTMS prior to initiation of treatment via brain-based biomarkers would be time- and cost-effective to patients and providers.

Biomarkers guiding rTMS prescription must be sensitive, specific, and reliable to be clinically useful. Resting-state functional connectivity, structural imaging (cortical thickness), positron emission tomography (PET), single photon emission computed tomography (SPECT), and various electroencephalography (EEG) metrics have identified candidate neuroimaging-based biomarkers of rTMS outcomes [17–21]. Examples include high glucose metabolism in the frontal lobe [21] and anticorrelation between connectivity of the subgenual cingulate cortex and the left dorsolateral prefrontal cortex (DLPFC) [22]. While they have pointed to changes that correspond with successful treatment, none to date has proven sufficient for clinical application given the existing lack of standardization in data collection, preprocessing, and analysis, invasiveness (PET), duration of protocol, and variability in findings [23, 24].

One candidate method deserving of further investigation is proton magnetic resonance spectroscopy (MRS), an imaging technique that measures the concentration of biochemical compounds in the brain in vivo [25]. MRS measures total *N*-acetylaspartate (tNAA), a combination of *N*-acetylaspartate (NAA) and *N*-acetylaspartylglutamic acid (NAAG), and glutamatergic compounds (Glx), comprised of glutamate (Glu) and glutamine (Gln). In comparison to other techniques, MRS has several advantages: it is short in duration (~3 mins), has relatively standardized data collection and preprocessing techniques [26], reliably quantifies metabolites throughout the brain [27, 28], and provides an accurate, non-invasive measure of cellular metabolism [29], contributing substantive insight on depression neuropathology.

Glutamatergic and *N-*acetylated compounds have been associated with multiple neuronal and glial processes implicated in MDD [30–34]. Glu is the most abundant excitatory neurotransmitter in the brain and, along with Gln, is thought to reflect neuronal energy metabolism [35]. tNAA serves as a potential marker of neuronal health and integrity [36, 37] and may mitigate glutamatergic excitotoxicity [38]. Glutamatergic system dysfunction has been associated with heightened MDD symptomatology, as low tricarboxylic acid cycle activity may lead to aberrant excitatory neurotransmission within and between frontal and limbic structures [39–41].

In comparison to healthy controls, individuals with MDD often exhibit lower concentrations of Glx, Glu, and tNAA in the anterior cingulate cortex (ACC) and prefrontal cortex (PFC) [40, 42–44]; low metabolite levels are also associated with heightened depression severity [40, 43, 45–47]. Indeed, baseline glutamatergic metabolites may be biomarkers of rTMS outcomes: the majority of existing studies show individuals with low baseline concentrations of frontal lobe Glu and Glx have the greatest symptom improvement post-rTMS [41, 48–50], while others indicate higher concentrations of Glx and Glx/tCr predict greater reduction of depression symptoms [51, 52]. More research is required to reconcile the directionality of these findings. Though the relationship between baseline tNAA and depression improvement following rTMS was not significant in one study [51], others found lower levels of baseline tNAA in the ACC predicted MDD recovery following electroconvulsive therapy (ECT) [53, 54]. Thus, further exploration is needed to evaluate *N*-acetylated compounds as biomarkers of rTMS outcomes.

Although low Glu and tNAA have been significantly linked to specific MDD symptom domains including mood and cognition [55–60], studies have primarily examined the relationship between baseline metabolite levels and overall depression symptom scores following rTMS [41]. This is a limitation of the current literature, as rTMS has demonstrated the ability to ameliorate a broad spectrum of symptoms [61–64]. Characterizing the relationship between pre-treatment metabolite levels and improvement in specific symptoms clusters following rTMS may be useful for elucidating transdiagnostic pathological processes involved in neuropsychiatric disorders, thereby guiding a personalized medicine approach to rTMS therapy.

In this exploratory study, we evaluated baseline levels of Glx, Glu, and tNAA in the right dorsal anterior cingulate cortex (dACC) as predictors of rTMS treatment outcomes in patients with depression. We chose the dACC, a core node of the salience network and limbic system, as our region of interest because of its involvement in attentional control and affective evaluation [65–67]. Given its role in mood and cognition, hypoactivation of this structure has been associated with heightened symptoms in MDD [68]. rTMS-associated increases in glutamatergic metabolism may strengthen neurotransmission within the dACC and its connections to other frontolimbic structures, ultimately improving attentional control over negatively valanced self-referential thoughts and decreasing rumination and low mood in MDD. Though the DLPFC is traditionally used as a region of interest in predicting rTMS outcomes because it is typically the site of stimulation, imaging studies reveal significant relationships between metabolic [41] and functional signals [69, 70] in the dACC and post-rTMS outcomes in individuals with depression, providing further support for our voxel placement. Our selection of the right dACC was motivated by the demonstrated feasibility of our previous work [71] and that right dACC morphology at baseline is predictive of clinical response to intermittent theta burst stimulation [72]. Additionally, research suggests both the right and left dACC are functionally connected to the rTMS target (left DLPFC) in individuals with MDD [73], implicated in depression pathology [74], and may yield similar results.

Based on the summarized findings, we anticipated that participants with lower levels of Glu, Glx, and tNAA would experience a greater reduction in overall depression severity following a standard course of rTMS. We also expected that lower baseline metabolite levels would predict improvement in mood and cognitive symptoms. This is based on prior work associating low Glu, Glx, and tNAA with greater symptom severity, and evidence indicating that rTMS appears to modulate neural activity in salience circuit nodes, such as the dACC, underlying depressed mood and cognitive dysfunction [75].

## Materials and methods

### Study Overview

**Fig. 1:**
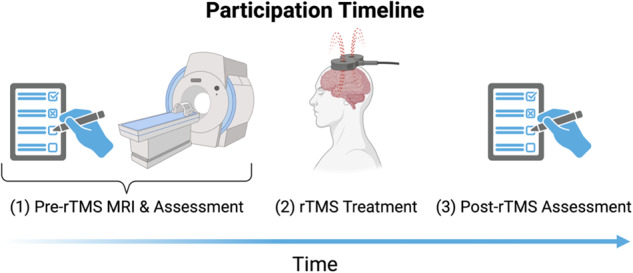
Participation Timeline. (1) The pre-rTMS MRI and assessment were completed approximately two weeks prior to the initiation of rTMS; (2) participants then underwent a full course of rTMS (an acute phase of 36 sessions with possible extension up to 46; see the rTMS procedures section and Table 1 for more details); (3) the post-rTMS assessment was completed on the last day of rTMS treatment. Created with biorender.com; rTMS: repetitive transcranial magnetic stimulation.

Informed consent was obtained from all participants. Study participants completed an eligibility and enrollment interview, a baseline MRI scan, and self-report symptom assessment approximately two weeks prior to beginning rTMS. If the first rTMS session occurred more than two weeks after baseline procedures, depression severity was reassessed prior to rTMS. Participants then completed standard rTMS treatment (once-daily sessions for 6 weeks followed by 6 sessions tapered over 3 weeks (36 sessions); the number of sessions could be extended by up to 10 (46 maximum sessions); see the rTMS procedures section for further details). Post-rTMS symptom assessment was completed at the final rTMS session. All participants gave permission for research use of symptom data collected during routine rTMS care. See Fig. 1.

rTMS treatment and all clinical assessments took place at the Butler Hospital Transcranial Magnetic Stimulation (TMS) Clinic in Providence, RI, USA. MRI scanning took place at the Brown Magnetic Resonance Imaging Facility in Providence, RI, USA. rTMS treatments represented a course of naturalistic, insurance-covered care; the collection of MRI and additional symptom data was supported by an NIH grant (MH113929, PI Michael Fox, MD, PhD). Procedures were approved by the Institutional Review Boards of Beth Israel Deaconess Medical Center, Butler Hospital, and the Mass General Brigham Human Research Committee of Brigham and Women’s Hospital.

### Participants

Participants (*n* = 27) with a primary diagnosis of MDD [76] and a history of resistance or intolerance to standard antidepressant medication were recruited from the Butler Hospital TMS Clinic after it was determined they were approved for insurance-covered treatment. Treatment resistance (and hence eligibility for treatment and study participation) was variably defined by different medical insurance policies of the patients presenting to the TMS clinic, and generally required at least two unsuccessful antidepressant medication trials (i.e., an ineffective trial despite adequate dose and duration, or a trial terminated at subtherapeutic does/duration due to intolerable side effects); some policies also required a past trial of psychotherapy to be eligible for rTMS insurance coverage. Eligible participants (1) were able to read and speak English; (2) were 18-80 years old; (3) had undergone an extensive evaluation wherein a psychiatrist established a primary diagnosis of nonpsychotic, recurrent MDD and a treatment history of antidepressant medication resistance or intolerance; (4) met rTMS safety criteria and all other eligibility criteria for rTMS therapy according to their insurance; (5) had no prior history of rTMS treatment; (6) had not received ECT within the current depressive episode or within the past three months; and (7) met safety criteria for MRI scanning. Ineligible participants had any (1) comorbid (nonprimary) or past psychiatric disorder, that in the judgement of the investigators, had symptoms severe enough to interfere with the validity of the data collected; (2) significant current neurological illness (Parkinson’s disease, dementia, intracranial pressure, etc.); and (3) history of a seizure disorder.

### Self-report assessments of MDD severity and symptom domains

MDD severity and symptom domains were measured using the Inventory of Depressive Symptomatology—Self Report (IDS-SR) scale [77], a standard 28-item measure [78] that has been consistently used to evaluate rTMS-associated outcomes [79, 80]. “Response” was categorically defined by 50% or greater reduction in IDS-SR total score from pre-treatment to post-rTMS (following the final session).

We analyzed the relationship between baseline metabolites and percent change from baseline to post-treatment in scores on three IDS-SR subscales developed by Han et al. (2021) [81]. These subscale domains represented “mood/cognition” (15 items; max score of 45), “somatic” (10 items related to bodily problems; max score of 30), and “immuno-metabolic” (5 items related to atypical/energy symptoms; max score of 15). Higher scores on the subscales indicated greater severity of symptoms in those dimensions. See [81] for further details regarding subscale construction.

As an exploratory analysis, we used principal component analysis (PCA) to reduce the dimensionality of the IDS-SR symptom domains (SPSS Statistics 28 (IBM, Armonk, NY, USA)). Because there is currently no expert consensus on which IDS-SR subscales best characterize MDD symptom domains, PCA demonstrated convergence between the data-driven grouping of the 30 individual items and the Han et al. (2021) subscales. We calculated percent change from baseline to post-treatment for the score on each IDS-SR item (((post – pre)/pre))*−100), and entered them into the PCA. 1 was added to each pre- and post-treatment score to avoid dividing by 0 in the percent change equation. An orthogonal varimax rotation with Kaiser normalization was applied during PCA. This yielded 30 factors, however, only the first two were utilized in subsequent analyses as they contributed to the majority of the variance (>10% each, Eigenvalue > 3). Items were retained for factor interpretation if the loading on the factor was >|0.3|.

### rTMS procedures

Participants underwent standard-of-care rTMS treatment, i.e., a 6-week course of once-daily (5/week) sessions followed by 6 sessions tapered over 3 weeks. In cases (*n* = 5) where permitted by insurance, the acute course of 36 sessions was extended by up to 10 sessions when the patient had late onset of response or had not achieved remission (maximum of 46 sessions). The acute phase may have been truncated, and the taper phase started prior to session #30 for patients showing sustained remission before that point. A minority were not taking any psychiatric medications during the course of rTMS. The majority of rTMS patients continued stable doses of concurrent antidepressant medications or had minor dosage changes during the course. In rare cases (*n* = 2) it was medically necessary to start a new antidepressant medication. Concurrent psychiatric medications included antidepressants, stimulants, antipsychotics, anxiolytics, hypnotics, and mood stabilizers. rTMS patients in the clinic all initiate their first treatment series with the standard 10 Hz protocol (3000 pulses per session), applied with a figure-8 coil that targeted the left DLPFC with stimulation at an intensity of 120% relative to resting motor threshold. Standard clinical treatment protocol at the Butler Hospital TMS clinic was used to identify scalp location for coil placement over the DLPFC target (5 cm anterior to the hand representation of the motor cortex) including head measurements via flexible measuring tape and administration of single TMS pulses. Minor modifications to a patient’s stimulation protocol (i.e., adjusting the frequency and to which hemisphere the stimulation was applied) were made if deemed medically necessary by the treating physician to manage tolerability or optimize outcomes. Clinical and demographic features of the sample, along with rTMS treatment details, appear in Table 1; the protocol delivered during the majority of treatments is reported as the dominant protocol. Treatments were delivered using a NeuroStar TMS Therapy system (Neuronetics, Inc., Malvern, PA, USA) or a Nexstim NBS device (Nexstim Ltd., Helsinki, Finland).

**Table 1:** Participant Demographic and Clinical Data.

Demographic & Treatment Characteristics	Mean (SD) or *n* (%)		
	Total Sample (*n* = 25)	Responders (*n* = 13)	Non-Responders (*n* = 12)
Age, mean (SD)	38.04 (14.57)	37.92 (14.40)	38.17 (15.40)
Women, *n* (%)	14 (56.00)	9 (69.20)	5 (41.70)
Race, *n* (%)			
White	24 (96.00)	12 (92.3)	12 (100.00)
Black or African American	0 (0.00)	0 (0.00)	0 (0.00)
Asian	1 (4.00)	1 (7.70)	0 (0.00)
Native Hawaiian or Pacific Islander	0 (0.00)	0 (0.00)	0 (0.00)
American Indian or Alaskan Native	0 (0.00)	0 (0.00)	0 (0.00)
Ethnicity, *n* (%)			
Hispanic or Latinx	1 (4.00)	0 (0.00)	1 (8.30)
Psychiatric Medications at Baseline, *n* (%)			
Antidepressants (SSRIs, SNRIs, tricyclics, etc.) *n* (%)	22 (88.00)	11 (84.60)	11 (91.70)
Benzodiazepines/Hypnotics	9 (36.00)	6 (46.20)	3 (25.00)
Antipsychotics	9 (36.00)	6 (46.20)	3 (25.00)
Stimulants	4 (16.00)	1 (7.70)	3 (25.00)
Total Number of rTMS Treatments, mean (SD; range)	37.48 (6.87; 15–46)	39.69 (8.87; 15–46)	35.08 (2.23; 29–36)
Number of Weeks Receiving rTMS Treatment, mean (SD)	9.75 (2.20)	10.33 (2.72)	9.13 (1.27)
Dominant rTMS Treatment protocol, *n* (%)			
10 Hz (left DLPFC)	17 (68.00)	7 (53.80)	9 (75.00)
5 Hz (left DLPFC)	2 (8.00)	3 (23.10)	0 (0.00)
1 Hz (right DLPFC)	4 (16.00)	3 (23.10)	1 (8.30)
Mixed Protocols	2 (8.00)	0 (0.00)	2 (16.70)
IDS-SR Full Scale, mean (SD)			
Pre-Treatment	43.20 (6.22)***	42.08 (6.18)***	44.42 (6.29)*
Post-Treatment	23.28 (14.19)***	11.85 (6.07)***	35.67 (8.79)*
Percent Change	46.35 (32.66)	72.23 (13.04)	18.32 (22.21)
IDS-SR Mood/Cognition Subscale, mean (SD)			
Pre-Treatment	27.92 (4.09)***	26.92 (3.93)***	28.42 (4.58)**
Post-Treatment	14.38 (9.45)***	7.31 (4.64)***	22.73 (6.15)**
Percent Change	49.68 (32.01)	73.65 (15.98)	21.35 (20.51)
IDS-SR Somatic Subscale, mean (SD)			
Pre-Treatment	9.83 (4.21)***	9.23 (4.85)***	10.42 (3.26)
Post-Treatment	5.54 (4.49)***	2.92 (2.14)***	8.64 (4.63)
Percent Change	42.76 (40.38)	64.74 (27.60)	16.78 (38.28)
IDS-SR Immuno-metabolic Subscale, mean (SD)			
Pre-Treatment	5.75 (2.75)***	5.54 (2.03)***	5.58 (3.65)
Post-Treatment	3.08 (2.32)***	1.62 (1.19)***	4.82 (2.14)
Percent Change	39.40 (50.83)	70.80 (23.95)	2.23 (50.56)

*p < 0.05; **p < 0.01; ***p < 0.001**;** the total sample for the IDS-SR Mood/Cognition Subscale, Somatic Subscale, and Immuno-metabolic Subscale only include 24 participants; the non-responder group has 11 participants for the IDS-SR Mood/Cognition Subscale, Somatic Subscale, and Immuno-metabolic Subscale data; as recommended by the National Institute of Health, race and ethnicity are considered separate categories and one person in the non-responder group identified as both white and Hispanic/Latinx.

*SSRIs* selective serotonin reuptake inhibitors, *SNRIs* serotonin-norepinephrine reuptake inhibitors, *rTMS* repetitive transcranial magnetic stimulation, *IDS-SR* Inventory of Depression Symptomatology Self-Report.

### MRI data collection procedures and analysis

Scanning was conducted on a Siemens (Erlangen, Germany) PRISMA 3 Tesla (T) MRI scanner (Siemens Medical Solutions, New York, NY, USA) and a 64-channel head coil.

#### Structural MRI

High resolution T1-weighted structural images were acquired from each participant via 3D-turbo field multiecho MEMPRAGE (slice thickness = 1 mm^3^, sagittal orientation, TR = 2530 ms, TE = 1.69 s, flip angle = 7°, in-plane matrix = 256^2^, slices = 176). Raw structural data was converted to the NIfTI-1 format using the Python-based program HeuDiConv (version 0.9.0) [82].

#### MRS

**Fig. 2:**
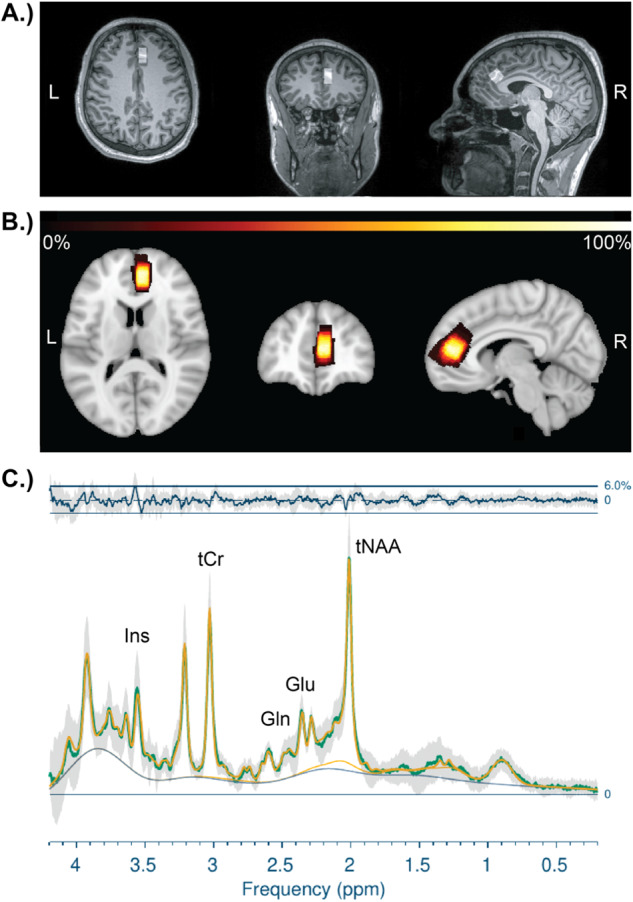
Voxel Placement and Spectra. **A** An example right dACC (15 x 15 x 10 mm^3^) voxel placed over its corresponding T1-weighted MEMPRAGE; **B** Composite right dACC voxel placement in all participants (*n* = 25), where the voxels shown are normalized to Montreal Neurological Institute (MNI)-152 space and the whiter the color, the greater percent of voxel overlap; **C** Average PRESS spectrum of all 25 participants, where the green line represents the average raw input data with no smoothing, the yellow line represents the average LCModel fit, and the gray shading represents standard deviation of the raw input data. The blue line at the top is a plot of the residual error, or the data minus the fit of the data, and the curved blue line underneath the spectra represents the baseline [85]. : *L* left, *R* right, *Ins* myoinositol, *tCr* total creatine, *Glx* glutamine and glutamate, *Glu* glutamate, *tNAA* total *N-*acetylaspartate.

A single voxel was collected in the right dACC (15 x 15 x 10 mm^3^) using standard single voxel Point RESolved Spectroscopy (PRESS) (TE = 30 ms, TR = 3000 ms, averages = 64), which reliably allows for quantification of Glu, Glx, tNAA, Ins, and tCr at 3 T [83–85]. Guided by our group’s previous work quantifying Glu, Glx, and tNAA [71, 86], the voxel was placed and tangentially rotated to be anterior and parallel to the corpus callosum; the posterior portion of the voxel was placed in line with the frontal horns of the lateral ventricle; cerebrospinal fluid (CSF) contamination was minimalized along the longitudinal fissure. See Fig. 2A for example placement and 2B for a composite image of participants’ voxels. First-order auto-shimming and manual shimming were implemented.

PRESS data were processed and quantified using LCModel (version 6.3-1R) with a basis set provided by LCModel [85], accounting for scanner-specific timing and pulse sequences. Automatic zero- and first-order phase correction was done by LCModel, and only metabolites with a Cramer-Rao lower bound (CRLB), or estimate of LCModel fit, less than or equal to 20% *SD* were evaluated [71]. See Fig. 2C for the average spectrum across all participants. Using Gannet CoRegister (version 3.1), voxel masks were coregistered to individuals’ T1-weighted images [87, 88], providing percentages of white matter, grey matter, and CSF in the voxel. Participants with less than 60% gray matter were excluded from analyses for quality control purposes [71]. In accordance with expert consensus and recommendations for MRS [26], data were then corrected for water- and metabolite-specific T1 and T2 relaxation constants and water density [89], and for partial volume effects of CSF using the equation [*1/(1- CSF)] [88, 90]. This yielded water-referenced molal concentrations of Glu, Glx, tNAA, Ins, and tCr. To evaluate the specificity of Glu, Glx, and tNAA, we also analyzed Ins and tCr as comparator metabolites given the lack of evidence linking them to MDD pathoetiology or rTMS outcomes [41].

### Statistical analysis

We excluded two participants with < 60% gray matter in the dACC voxel. Item-level IDS-SR data was unavailable for one participant. Thus, IDS-SR subscale and PCA analyses were based on *n* = 24 participants, whereas analyses involving overall IDS-SR scores had *n* = 25. We used Python 3 [91] or SPSS Statistics 28 for statistical analyses. We calculated descriptive statistics for baseline demographic and clinical data, rTMS treatment parameters (number of treatments, number of weeks receiving treatment, dominant rTMS treatment protocol), IDS-SR total and scores for the PCA symptom factors, as well as mood/cognition, somatic, and immuno-metabolic subscales. Descriptive statistics also characterized number of rTMS responders, IDS-SR percent change (total and subscale scores), and baseline metabolite levels.

We used Shapiro-Wilk tests to assess the normality of metabolite levels and depression score distribution. If distributions violated assumptions of normality (Shapiro-Wilk *p* < 0.05) they were sigmoidally transformed and the transformed variable was used in all subsequent analyses. Two-tailed Pearson correlations were used to evaluate CSF dependence via CSF voxel percentage and raw Glu, Glx, tNAA, Ins, and tCr molal concentrations (no tissue correction). Two-tailed paired t-tests compared pre- and post-rTMS IDS-SR scores. Independent samples t-tests and chi-square tests were conducted to compare responders versus non-responders on baseline IDS-SR full score, PCA and subscale scores, total number of rTMS sessions received, age, and sex. Independent samples t-tests were also used to test for baseline equivalence between response groups in the fraction of dACC voxel gray matter, white matter, and CSF.

Generalized linear regression models were used to test relationships between baseline metabolite levels and percent change in depression symptom severity (full IDS-SR) or change in specific symptom subdomains. Percent change scores were used to evaluate continuous rTMS outcomes and accounted for baseline MDD severity. This was done by subtracting the pre-treatment score from the post-treatment score, and then dividing that value by the pre-treatment score. The resultant decimal was multiplied by −100 to provide a value between −100 and 100, where positive numbers indicated symptom improvement, 0 indicated no change, and negative numbers indicated worsening of symptoms (equation: ((post– pre)/pre))*−100). This method is commonly used when evaluating the effects of metabolites on rTMS outcomes [50, 52, 92]. Separate models were created for each metabolite predicting percent change in score on six symptom scales (the IDS-SR full scale, 2 PCA-derived component scales, and the 3 Han et al. (2021) published subscales: mood/cognition, somatic, and immuno-metabolic) from pre-treatment metabolite level (continuous variables). We included age as a covariate in all models given the negative association of age with metabolite levels [93–95]. Sex was also included as a covariate in the models given reported sex differences in MDD etiology and symptom presentation [96], rTMS outcomes [97], and glutamatergic depression correlates [98]. In total, we created 18 generalized linear regression models for primary hypothesis testing, one for each metabolite (x3) and outcome (x6). Additional models for Ins and tCr were constructed in the same fashion to evaluate the specificity of hypothesized effects of interest. Bivariate Pearson’s correlations for Glu, Glx, and tNAA were plotted for illustrative purposes only as the resulting *p*-values are not independent [99].

Additional logistic regressions were conducted to evaluate baseline metabolites as predictors of binary outcomes, i.e., rTMS treatment response. There was a total of five models, i.e., 3 for the metabolites of interest and 2 metabolites for evaluating specificity. Age, sex, and baseline total IDS-SR scores were also included as covariates.

A Bonferroni correction (*p* ≤ 0.007) was applied for the seven outcomes of interest: change in IDS-SR total score, PCA Factor 1 score, PCA Factor 2 score, and scores on the mood/cognition, somatic, and immuno-metabolic subscales; and the dichotomous responder status. Following our previously published work, each metabolite was treated as its own family of tests [71]. Generalized linear regression and logistic regression outcomes reported in the Results are Bonferroni-corrected; any outcomes between *p* = 0.008-0.05 are considered to be marginally significant. Relationships between metabolites and outcomes were tested for influence of outliers via Cook’s *D*. Post-hoc sensitivity tests were conducted on models where change in psychiatric medication status (change=1, no change=0) and dominant rTMS protocol (excitatory (>1 Hz, over left DLPFC) = 1 or inhibitory (1 Hz over right DLPFC) = 0) were included as independent covariates in separate models to ensure they did not influence metabolite relationships with rTMS outcomes.

The area under the receiving operating characteristic (AUROC) curve [100] was computed for each metabolite (Glu, Glx, tNAA, Ins, and tCr) and the sensitivity (true positive rate) and specificity (true negative rate) were evaluated for prediction of responder status. We used the Hosmer-Lemeshow goodness of fit test to assess our models’ discriminative abilities [101]. The significance of the assessed performance predictions was not subjected to the Bonferroni correction.

## Results

### Participant characteristics and clinical outcomes

Our sample (*n* = 25) was primarily Caucasian (96%, *n* = 24) and non-Hispanic (96%, *n* = 24), with an average age of 38 years (*SD* = 14.57); approximately 56% (*n* = 14) identified as females. Baseline IDS-SR scores were classified as “severe” with an average of 43.20 (*SD* = 6.22). Participants received an average of 37.48 (*SD* = 6.87) rTMS sessions over 9.75 (*SD* = 2.20) weeks. The average time between the MRI/self-report and rTMS start was 13.75 days (*SD* = 15.61). Seventeen (68%) were treated with predominantly left DLPFC 10 Hz stimulation, 2 (8%) with left DLPFC 5 Hz, 4 (16%) with right DLPFC 1 Hz, and 2 (8%) with mixed protocols, meaning they had 50% of one protocol and 50% of another. Following the final treatment session, 13 (52%) participants were classified as rTMS responders (Table 1).

The mean IDS-SR (full scale) score significantly decreased from baseline to treatment endpoint (*p* < 0.001), reflecting an average 46.35% decrease (*SD* = 32.66). Similarly, there were significant decreases in the mood/cognition, somatic, and immuno-metabolic subscales (*p* < 0.001). Shapiro-Wilk tests determined all percent change scores were normally distributed (*W*(24) = 0.92–94; *p* = 0.05–13). When comparing treatment responders and non-responders at baseline, there were no significant differences between mean IDS-SR scores (full-scale and subscales), mean number of rTMS sessions received, age, and sex (*p* > 0.05).

### Baseline Metabolite Levels

Means and standard deviations for baseline Glu, Glx, tNAA, Ins, and tCr levels and their corresponding CRLBs are in Table 2. Shapiro-Wilk tests determined that all metabolite values other than tCr were normally distributed (*W*(25) = 0.93-0.98 ; *p* > 0.05). Originally, tCr had a mean of 6.06 institutional units (*SD* = 0.76) and was non-normally distributed *W*(24) = 0.72, p < 0.00001; following sigmoidal transformation, the mean was 1.00i.u. (*SD* = 0.00), and was normally distributed *W*(25) = 0.95 (*p* = 0.31). Tissue percentages of gray matter, white matter, and CSF are presented in Table 2. There were no significant relationships between raw metabolite concentrations and CSF dependence (all *p* > 0.05). All CRLB averages and full width half maximum (FWHM) values (Table 2) were within the standards for collecting high quality spectroscopy data [85]. There were no significant group differences in tissue percentages between rTMS responders and non-responders (*p* > 0.05).

**Table 2:** Metabolite Data.

Metabolite Characteristics	Mean (SD)		
	Total Sample (*n* = 25)	Responders (*n* = 13)	Non-Responders (*n* = 12)
Metabolite, mean (SD)			
Glu	8.76 (1.05)	8.24 (0.87)	9.33 (0.94)
Glx	11.14 (1.30)	10.61 (1.05)	11.71 (1.34)
tNAA	7.23 (0.82)	6.95 (0.40)	7.54 (1.05)
Ins	4.52 (0.63)	4.42 (0.56)	4.62 (0.71)
tCr	1.00 (0.00)	1.00 (0.00)	1.00 (0.00)
FWHM, mean (SD)	16.14 (2.10)	16.50 (2.03)	15.75 (2.20)
CRLB, mean (SD)			
Glu	6.28 (0.98)	6.38 (1.12)	6.17 (0.84)
Glx	5.80 (0.76)	5.85 (0.80)	5.75 (0.75)
tNAA	3.72 (0.68)	3.62 (0.65)	3.83 (0.72)
Ins	4.72 (0.61)	4.62 (0.51)	4.83 (0.72)
tCr	2.96 (0.54)	2.77 (0.44)	3.17 (0.58)
% Voxel Composition, mean (SD)			
Gray Matter	69.68 (5.03)	69.35 (4.53)	70.04 (5.72)
White Matter	13.85 (5.57)	15.08 (6.25)	12.52 (4.60)
CSF	16.47 (6.51)	15.57 (7.62)	17.44 (7.42)

Sigmoidally transformed tCr values are reported in table for mean metabolite level; *Glu* glutamate, *Glx* glutamate and glutamine, *tNAA* total *N*-acetylaspartate, *Ins* myoinositol, *tCr* total creatine, *FWHM* full width half maximum, *CRLB* Cramer-Rao Lower Bound.

### Principal Component Analysis

The PCA (*n* = 24) yielded 2 factors meeting a priori thresholds. The first, which we call the “PCA mood & cognition factor,” had an Eigenvalue of 8.81 (29.35% of variance) and 22 item loadings. The second, which we refer to as the “PCA somatic factor,” had an Eigenvalue of 3.726 (12.42% of variance) and 16 item loadings. The factors were not significantly correlated (*p* > 0.05), justifying the orthogonal varimax rotation. See Table 3 for individual item loadings.

**Table 3:** Item loadings for 2 PCA Factors and Inclusion in Han et al. (2021) Published Subscales.

Percent Change in IDS-SR Item	PCA Factors		Han et al. (2021) Subscales		
	Mood & Cognition	Somatic	Mood/Cognition	Somatic	Immuno-Metabolic
Difficulty falling asleep		0.547		X	
Awakening from sleep during the night	0.503			X	
Waking up too early		0.314		X	
Sleeping too much					X
Feeling sad	0.803		X		
Feeling irritable	0.46	−0.358	X		
Feeling anxious or tense	0.659		X		
Mood Reactivity to good or desired events	0.78		X		
Diurnal variation		0.723	X		
Quality of mood	0.719		X		
Decreased appetite		−0.65		X	
Increased appetite		0.373			X
Weight loss	0.36			X	
Weight gain		0.434			X
Impaired concentration/ decision making	0.842		X		
Low view of self	0.746	−0.377	X		
Pessimism towards future	0.602	−0.372	X		
Thoughts of death or suicide	0.481	−0.325	X		
Loss of general interest	0.765		X		
Loss of energy	0.836				X
Loss of capacity for pleasure	0.677		X		
Loss of libido	0.471	0.317	X		
Feeling slowed down	0.596			X	
Feeling restless	0.468			X	
Aches and pains		0.613		X	
Other bodily symptoms	0.377	0.621		X	
Panic/ phobic symptoms	0.456	0.417	X		
Constipation/diarrhea	0.474	0.31		X	
Interpersonal sensitivity	0.473	−0.413	X		
Leaden paralysis/ physical energy	0.715				X

Item loadings for the two factor PCA outcomes (*n* = 24); individual items were entered as percent change scores from baseline to post-treatment. Only loadings where >|0.3| are shown. Factor 1 is characterized as the mood & cognition factor, while factor 2 is characterized as the somatic factor.

*IDS-SR* Inventory of Depression Symptomatology Self-Report, *PCA* principal components analysis.

### Predictors of Treatment Outcomes (Continuous Variables)

#### Percent Change in IDS-SR Total Score

**Fig. 3:**
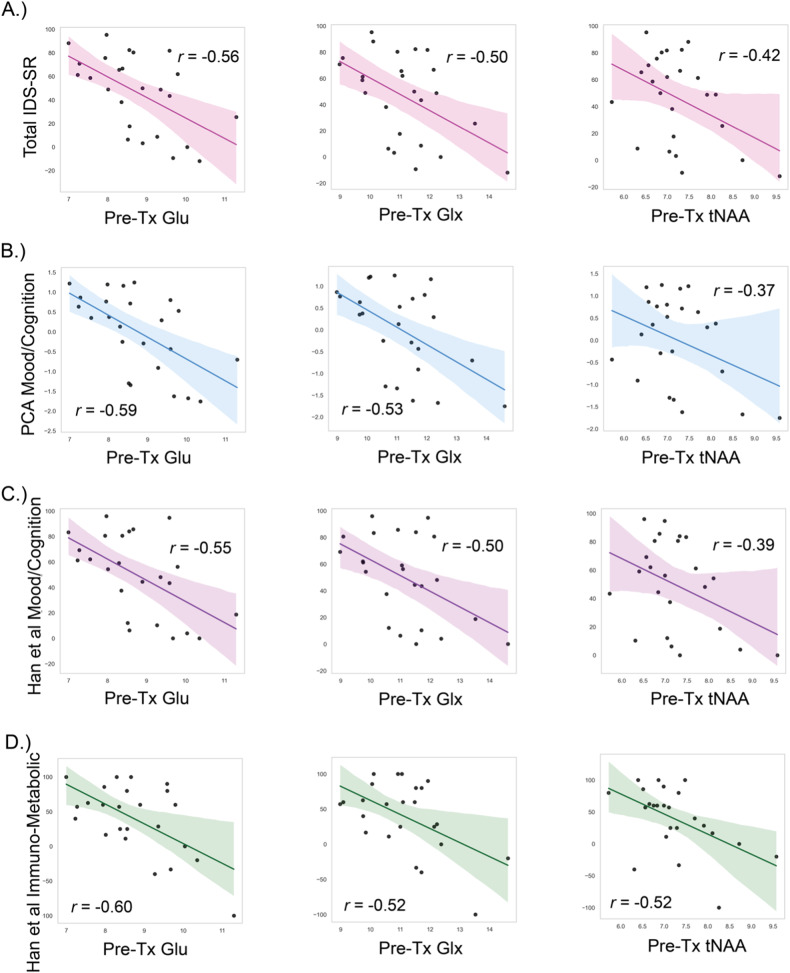
Metabolites as Predictors of Percent Change in IDS-SR Full Scores and Symptom Domains following rTMS. Scatter plots of bivariate Pearson correlations between baseline metabolites and continuous treatment outcomes. Panels show: **A** percent change in full scale IDS-SR score and Glu, Glx, and tNAA (*n* = 25); **B** PCA mood & cognition factor scores and Glu, Glx, and tNAA (*n* = 24); **C** percent change in the IDS-SR mood/cognition subscale and Glu, Glx, and tNAA (*n* = 24); and **D** percent change in the IDS-SR immuno-metabolic subscale and Glu, Glx, and tNAA (*n* = 24). Greater percent change indicates greater improvement in symptoms; *p*-values are not shown as noted in the text to avoid the “double-dipping” error of statistical significance. *IDS-SR* Inventory of Depression Symptomatology Self Report, *Glu* glutamate, *Glx* glutamine and glutamate, *tNAA* total *N*-acetylaspartate, *Pre-Tx* pre-rTMS treatment, *PCA* principal component analysis.

As hypothesized, lower levels of glutamatergic and *N*-acetylated metabolites at baseline were associated with greater improvement in overall depression severity (Fig. 3A). We observed significant effects of Glu (*B* = −17.17, *SE* = 4.96, *X*^*2*^ = 11.97, *p* < 0.001), Glx (*B* = −15.51, *SE* = 4.46, *X*^*2*^ = 12.106, *p* < 0.001), and tNAA (*B* = −21.73, *SE* = 8.02, *X*^*2*^ = 7.34, *p* = 0.007). Covariate effects were not significant, save a marginal effect of age in the Glx model (*B* = 0.835, *SE* = 0.39, *X*^*2*^ = 4.59, *p* = 0.032). Effects of metabolite were non-significant in Ins and tCr models.

#### Percent change in PCA-derived mood/cognition and somatic component scales

Lower pre-rTMS concentrations of Glu (*B* = −0.55, *SE* = 0.17, *X*^*2*^ = 13.72, *p* < 0.001) and Glx (*B* = −0.51, *SE* = 0.13, *X*^*2*^ = 15.159, *p* < 0.001) were also associated with greater improvement in mood/cognitive symptoms (PCA Factor 1; Fig. 3B). The effect of age was marginally significant in the Glx model (*B* = 0.03, *SE* = 0.01, *X*^*2*^ = 5.76, *p* = 0.016). Lower tNAA concentrations were associated with greater mood/cognition symptom improvement at marginal significance (*B* = −0.62, *SE* = 0.25, *X*^*2*^ = 5.89, *p* = 0.015). Effects of metabolite on mood/cognition were non-significant for the remaining models. Aside from a marginally significant effect of age in the Glx model (*B* = −0.03, *SE* = 0.01, *X*^*2*^ = 4.896, *p* = 0.027), no metabolites were significant predictors of change in somatic symptoms on the PCA somatic scale.

### Percent change in Han et al. [81] mood/cognition subscale

Low baseline Glu (*B* = −16.32, *SE* = 4.89, *X*^*2*^ = 11.15, *p* < 0.001) and Glx (*B* = −14.26, *SE* = 4.46, *X*^*2*^ = 10.25, *p* = 0.001) significantly predicted percent change in the published mood/cognition subscale (Fig. 3C). Lower tNAA concentrations were associated with greater mood/cognition symptom improvement at marginal significance (*B* = −18.61, *SE* = 8.09, *X*^*2*^ = 5.28, *p* = 0.022). Effects of metabolite on mood/cognition were non-significant for the remaining models.

### Percent change in Han et al. [81] somatic subscale

Low Glu (*B* = −14.52, *SE* = 7.13, *X*^*2*^ = 4.14, *p* = 0.042), Glx (*B* = −14.21, *SE* = 6.31, *X*^*2*^ = 5.07, *p* = 0.024), and tNAA (*B* = −24.66, *SE* = 10.53, *X*^*2*^ = 5.48, *p* = 0.019), predicted percent change on the somatic IDS-SR subscale with marginal significance. Effects of metabolite on the somatic subscale improvement were not significant for the remaining models.

### Percent change in Han et al. [81] immuno-metabolic subscale

Low Glu (*B* = −27.81, *SE* = 7.77, *X*^*2*^ = 12.87, *p* < 0.001), Glx (*B* = −22.26, *SE* = 7.38, *X*^*2*^ = 9.10, *p* = 0.003), and tNAA (*B* = −37.51, *SE* = 12.39, *X*^*2*^ = 9.17, *p* = 0.002) significantly predicted percent change in the immuno-metabolic subscale (Fig. 3D). Effects of metabolite on immuno-metabolic subscale percent change were not significant for the tCr and Ins models.

All relationships between metabolites and outcomes were not driven by outliers (*D* < 1). Post-hoc sensitivity analyses showed no significant effect of change in psychiatric medication status or dominant rTMS protocol on the relationship between metabolites and rTMS outcomes.

### Predictors of rTMS response (Dichotomous outcome)

The effect of baseline Glu level in the prediction of rTMS treatment response was marginally significant (*B* = 1.55, *SE* = 0.69, *X*^*2*^ = 5.04, *p* = 0.025). Effects of metabolites on responder status were not significant for the remaining models. Post-hoc sensitivity analyses showed no significant effect of change in psychiatric medication on the relationship between metabolites and rTMS response. Dominant rTMS protocol was not significant in any models, however, when entered in the Glx model, Glx (*B* = 1.46, *SE* = 0.68, *X*^*2*^ = 4.60, *p* = 0.032) became marginally significant when predicting responder status.

**Fig. 4:**
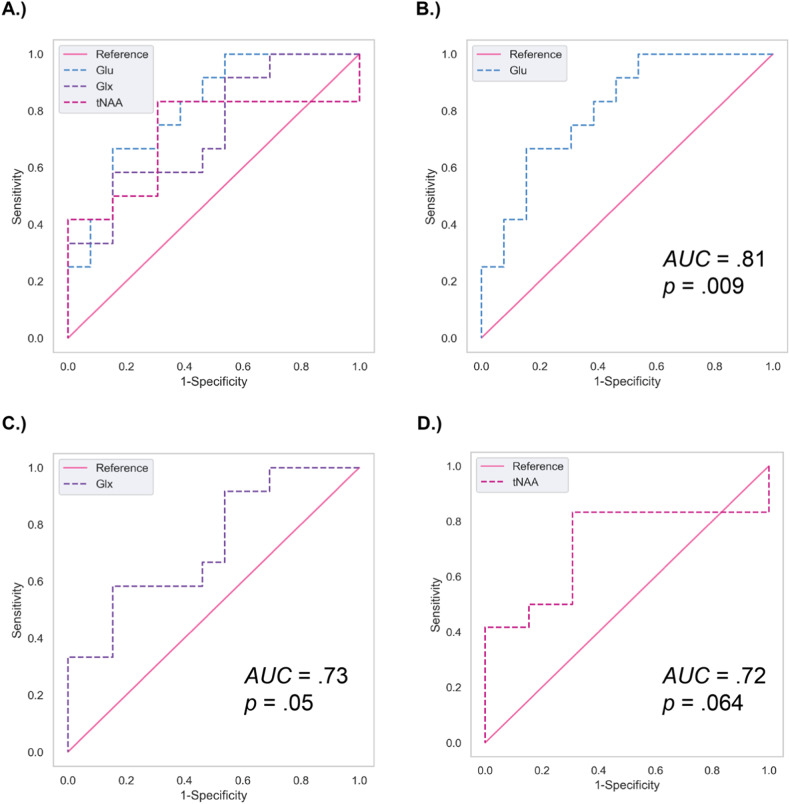
AUCs for Glu, Glx, and tNAA as predictors of rTMS Treatment Response. Area under the receiving operating characteristic (AUROC) curves for **B** Glu, **C** Glx, and **D** tNAA’s ability to predict rTMS responder status as a binary outcome (50% or greater improvement on full-scale IDS-SR score) from our generalized logistic regression analyses. Panel **A** shows how all three curves overlap with one another. The higher the AUC value, the greater the predictive ability of the baseline metabolite. *IDS-SR* Inventory of Depression Symptomatology Self Report, *Glu* glutamate, *Glx* glutamine and glutamate, *tNAA* total *N*-acetylaspartate.

The assessed performance prediction (Fig. 4) of the Glu model was excellent with an area under the curve (AUC) of 0.81 (*SE* = 0.09; *p* = 0.009). The performance for Glx and tNAA was acceptable, with AUC of 0.73 for Glx (*SE* = 0.10, *p* = 0.05) and AUC of 0.72 for tNAA (*SE* = 0.11, *p* = 0.064). Ins and tCr had no discriminative ability, with AUCs of 0.43 and 0.52 (*SEs* = 0.12, 0.12; *p* > 0.05).

## Discussion

This study examined Glu, Glx, and tNAA as potential, prospective pre-treatment biomarkers of response and change in symptom domains in adults with primary MDD who received naturalistic rTMS treatment. We found at baseline, (1) low Glu, Glx, and tNAA were predictive of greater post-rTMS change in overall depression severity; (2) low Glu and Glx were predictive of greater change in the mood/cognition domain; (3) low Glu, Glx, and tNAA were predictive of greater change in the immuno-metabolic domain; and (4) lower concentrations of Glu were predictive of rTMS responder status. These relationships were largely independent of age and sex, aside from a marginally significant contribution of age in the models evaluating the impact of Glx on mood & cognition and full IDS-SR score. As hypothesized, relationships were specific to glutamatergic and *N*-acetylated compounds, as both Ins and tCr were not predictive of percent change in symptomatology or rTMS responder status. These results indicate that Glu, Glx, and tNAA could serve as specific biomarkers predictive of outcomes a patient might expect following a full course of rTMS. For example, individuals with MDD who have relatively lower levels of such metabolites, i.e., Glu, may serve as the best candidates for rTMS.

rTMS outcomes were superior for individuals with low concentrations of frontal Glu prior to treatment, suggesting rTMS may be most effective when upregulation of the Glu-Gln cycle can occur. The glutamate hypothesis of depression suggests low intracellular Glu contributes to MDD pathology [39]. In our working model of rTMS mechanisms, we propose frontal lobe Glu-Gln dynamics are key mediators of the therapeutic effects of rTMS via upregulation of low neuronal and astrocytic Glu via increased glucose utilization and glycolytic pathway and tricarbolic acid (TCA) cycle activity [41]. We hypothesize that MDD symptoms thus remit to the extent that rTMS modulates the Glu-Gln pathway [41]. Given our model and current findings, we hypothesize that individuals who present with depression and low right dACC Glu concentrations are able to achieve greater post-rTMS clinical outcomes because neurons can successfully upregulate Glu production via the Glu-Gln pathway. This in turn leads to Glu levels that are optimal for cellular metabolism, neuronal plasticity, homeostasis of ATP production, and prevention of hyperammonia [102, 103]. In contrast, individuals with higher Glu levels when depressed may not respond as well to rTMS because they are already at ceiling-level. In these individuals, homeostatic, endogenous neuroprotective mechanisms that are already engaged may prevent Glu levels from rising too high to halt excitotoxicity and dynamic circuit imbalance [104].

As predicted, we found relatively lower Glu and Glx concentrations related specifically to percent change (i.e., more improvement) in mood and cognition-related symptoms. This is consistent with prior literature indicating that dACC hypoactivity is predictive of improved cognitive control following treatment in individuals with MDD [105]. Interestingly, there were also significant associations between Glu, Glx, and tNAA and percent change in immuno-metabolic symptoms, which included oversleeping, overeating, weight gain, decreased energy level, and leaden paralysis. Such neurovegetative symptoms are also robustly associated with salience network activity involving connections within and between the ACC, amygdala, and insular cortex in individuals with MDD. Together, the major nodes of the salience network, the insula and ACC, integrate information regarding sleep, digestive system activity, and are significantly influenced by hunger-related hormones such as leptin and insulin [106], providing additional support for the patterns observed here. Our findings that dACC Glu, Glx, and tNAA concentrations were not significantly related to the somatic symptom domain is consistent with prior work, which indicates that somatic symptoms are primarily associated with abnormal connectivity between the insula and regions involved in somatosensation (such as the precuneus, midcingulate, and angular gyrus) [107].

Though lower baseline levels of both Glu and Glx were related to rTMS-associated symptom improvement, Glx did not have as strong of an effect as Glu in the generalized linear models, and did not predict rTMS response as a binary outcome. Because Glu comprises the majority of the Glx signal, the observed relationships may be driven by Glu rather than Gln concentrations. This suggests (1) Gln may not be as mechanistically involved in rTMS-related MDD symptom improvement as Glu, or (2) baseline Gln levels in MDD may be heightened, rather than low like Glu, altering the directionality of metabolite-symptom effects. Gln should be collected at a higher field strength, as 3 T does not allow for reliable Gln quantification. Age’s marginal significance in the Glx models of mood/cognition and overall depression symptom improvement could be explained through the positive relationship between Gln and age [108]. tNAA may not have had as strong as an effect as Glu considering it is a general marker for neuronal health and integrity and serves as a precursor to for both Glu and Gln [36].

There are several limitations to this study. ^1^H MRS data were only collected from the right dACC; thus our metabolic findings may not generalize to the left hemisphere or other cortical regions. Relationships between baseline Glu, Glx, and tNAA and treatment outcomes may reflect a general improvement in depression symptomatology rather than a specific effect of rTMS. This may also be true for other noninvasive brain stimulation interventions, such as ECT. However, it is noteworthy that our sample was characterized by individuals who had not improved with standard antidepressant medications. Because our study is limited by a small sample size (*n* = 25) and underpowered to detect medium-to-small effect sizes, our primary objective was to establish feasibility of using baseline metabolites to predict rTMS outcomes. Our results are therefore considered preliminary and set the stage for future studies with larger sample sizes to detect small effect sizes. Sample homogeneity with regards to race and ethnicity inhibits our ability to generalize results to Black, Hispanic, Asian, and Indigenous individuals, and reflects systemic barriers to healthcare access across the US [109]. Future studies need to collect more data to understand how comorbid diagnoses (i.e., post-traumatic stress disorder), transdiagnostic symptoms (i.e., anxiety), number and type of previous treatments, and recurrence and previous history of MDD influence baseline metabolite levels and their relationships with rTMS outcomes in individuals living with MDD. The absence of this information may skew results and hinder replicability.

Our study has several methodological and analytical strengths. Data were collected prospectively, and were not subject to recall or retrospective selection bias. By analyzing baseline tCr and Ins, we demonstrate the metabolic specificity of relationships, and show that our Glu, Glx, and tNAA findings are not a product of overall metabolic upregulation or MRS scanning artifacts, such as poor water-suppression. We also evaluated (1) adults aged across the lifespan with (2) relatively equal representation of both sexes in the sample, facilitating generalizability.

Importantly, this study used a sample of individuals receiving standard rTMS therapy, including high frequency stimulation to the left DLPFC and low frequency stimulation to the right DLPFC. Differences in stimulation type and laterality may limit our ability to isolate mechanisms specific to either technique. However, the heterogeneity of rTMS treatment increases the ecological validity of our findings considering both high and low frequency rTMS are commonly implemented in clinical practice [12] and generally demonstrate equal efficacy [110]. Glutamatergic and *N*-acetylated metabolites may be non-specific biomarkers of outcomes for all rTMS techniques. The lack of effect of stimulation type in our models suggests different modes of rTMS may work via a common mechanistic pathway between the bilateral DLPFC and right dACC. The DLPFC and dACC are functionally and structurally connected [111], and rTMS to the DLPFC restores aberrant hypoconnectivity between these regions in individuals with MDD [73]. rTMS-induced depolarizations in the bilateral DLPFC may influence metabolic activity in the right dACC via glutamatergic projections between these regions, ultimately improving depression symptoms. More controlled research will be needed to evaluate any relationships between baseline metabolites and differential effects of stimulation protocol on depression outcomes.

Future work should include additional validated measures of specific MDD symptoms, such as depressed mood, anhedonia, comorbid anxiety, insomnia, impaired cognition, suicidality, and psychomotor agitation, to illuminate how Glu, Glx, and tNAA predict domains of symptomatic response. Additionally, future work should collect metabolite levels over multiple time points during and following rTMS treatment, enabling clinicians and scientists to better understand how rTMS influences metabolites longitudinally, and how changes in these levels correspond with treatment outcomes. With larger sample sizes, increased number of voxels per participant, information on symptom-specific outcomes, and metabolite concentrations over time, machine learning techniques could be used to better predict which individuals and depression biotypes [112] most likely to respond to rTMS, paving the way for an MRS-driven personalized medicine approach to rTMS therapy for MDD.

In summary, we found that pre-treatment levels of glutamatergic and *N*-acetylated compounds in the right dACC may serve as potential biomarkers for MDD outcomes after a full course of rTMS. Among MDD patients, lower levels of Glu, Glx, and tNAA predicted greater improvement in full scale depression scores after rTMS. Low baseline glutamatergic metabolites also specifically predicted greater decreases on symptom-specific changes in mood and cognition. Similarly, low Glu, Glx, and tNAA predicted decreases in scores on the immuno-metabolic subscale. Low Glu uniquely predicted rTMS responder status as a binary outcome. Together, these preliminary results provide rationale for exploring the utility of dACC Glu, Glx, and tNAA as biomarkers of rTMS efficacy for MDD. Though our findings need to be replicated with a larger sample size, this study is an important first step in (1) understanding how MRS metabolites predict and relate to rTMS outcomes associated with specific MDD symptom domains and (2) using neuroimaging-based techniques for optimization of psychiatric treatment selection, benefitting both patients and providers.

## Data Availability

The data that support the findings of this study are available from the corresponding author, MAG, upon reasonable request.
